# Pressure in the Treatment of Wounds

**Published:** 1898-12-17

**Authors:** 


### PRESSURE IN THE TREATMENT OF WOUNDS.

The discussion at a recent meeting of the Medical Society on the value of pressure in the treatment of wounds was of considerable utility, in that it brushed aside many cobwebs which have grown around this most ancient proceeding. Pressure has been used for a variety of purposes, and it has acted in a variety of ways—not always perhaps those that were intended—and we can hardly doubt that Mr. Pearce Gould is right in saying that not infrequently it has done harm. Where, for example, there is a large flap, as after the removal of a mamma, any such pressure as is likely to be effectual in restraining hæmorrhage may readily cause sloughing of the flap itself. Again, if pressure is used, as Mr. Gould says it is, to maintain apposition of the edges of the wound, it will be likely enough to be followed by the condition he describes, namely, union of the edges, while fluid collects beneath the flap. Pressure, however, is mostly employed in first dressings with the view of ensuring contact between the deeper parts of the wound, and this, we take it, is its real value. If pressure for a few hours can prevent the deeper portions of the wound becoming separated from each other by a layer of blood clot, and if the wound is so situated that such a moderate and regulated amount of pressure as will not interfere with the circulation will effect this object, we do not think that any of the facts brought forward in the discussion militate materially against its use; indeed, the very fact that Mr. Gould has had to return to the use of the drainage tube (to be left in for the first 24 or 48 hours) would tend to suggest that the abolition of pressure has been accompanied by an increased tendency to oozing. When, however, we turn to such illegitimate uses of pressure as its employment to prevent the bagging of pus, most surgeons will agree with Mr. Gould that it is not pressure, but a counter-opening that is required. It might have been as well to have drawn a distinction more markedly than was done between the use of pressure in regard to the wound and its use for other purposes, such as to hold back the viscera in case of vomiting after inguinal colotomy. Of course, in such a case the object of the pressure has nothing to do with its influence on the wound, and the propriety of using it depends upon how far in the particular case it is likely to effect the purpose for which it is employed. It can but rarely be desirable, for example, to exercise pressure upon a corpulent abdomen, the whole shape of which is altered on coughing or vomiting, the contracting muscles sometimes actually drawing the wound away from the pad which has been placed to press upon it. The inutility of such proceedings can be demonstrated any day if one attempts to hold in an umbilical hernia, in the case of a fat woman, by means of an ordinary bandage. But the case is quite different with the "mountains of cotton wool" to which Mr. Gould sarcastically refers as being still largely used in dressing cases of laparotomy for the removal of large ovarian cysts. These "mountains of cotton wool" are used for a definite purpose, not to exercise pressure, but to enable the bandage to occupy that form (namely, the form of a circle) which will best enable it to prevent the stretching of the flaccid abdominal parietes by the uncontrolled contraction of the diaphragm. If it will effect this purpose, as it mostly will in thin women, by all means let us use it. But, we repeat, a broad distinction must be drawn between the effect of pressure on a wound, which if it holds the deeper portions together surface to surface is good, and its effect in protecting a wound from mechanical interference. It is only very occasionally that it can be efficacious for the latter purpose, and, that being so, sutures and collodion will often be found more useful and far more comfortable than the ponderous bandagings sometimes employed.

